# *In vivo* evaluation of Sono-chemo therapy via hollow gold nanoshells conjugated to mitoxantrone on breast cancer

**DOI:** 10.22038/IJBMS.2023.67602.14811

**Published:** 2023-03

**Authors:** Vahid Abolzadeh, Armin Imanparast, Hooriyeh Nassirli, Naser Tayebi Meybodi, Bahareh Khalili Najafabad, Ameneh Sazgarnia

**Affiliations:** 1 Medical Physics Research Center, Mashhad University of Medical Sciences, Mashhad, Iran; 2 Department of Medical Physics, Faculty of Medicine, Mashhad University of Medical Sciences, Mashhad, Iran.; 3 Pharmaceutical Research Center, Institute of Pharmaceutical Technology, Mashhad University of Medical Sciences, Mashhad, Iran; 4 Department of Pathology, School of Medicine, Mashhad University of Medical Sciences, Mashhad, Iran

**Keywords:** Breast cancer, Chemotherapy, Combination therapy, Hollow gold nanoshell, Mitoxantrone, Sonodynamic therapy

## Abstract

**Objective(s):**

Conventional methods of cancer treatment include surgery, chemotherapy, radiation therapy, and immunotherapy. Chemotherapy, as one of the main methods of cancer treatment, due to the lack of targeted distribution of the drug in tumor tissues, is not able to destroy cancer cells and also affects healthy tissues and causes serious side effects in patients. Sonodynamic therapy (SDT) is a promising strategy for non-invasive treatment of deep solid cancer tumors. In this study, for the first time, the sono-sensitive activity of mitoxantrone was investigated and then mitoxantrone (MTX) was conjugated to hollow gold nanostructure (HGN) to improve the efficiency of *in vivo* SDT.

**Materials and Methods:**

Firstly, after the synthesis of hollow gold nanoshells and the PEGylation process, conjugation of MTX was performed. Then, after evaluating the toxicity of the treatment groups *in vitro*, in order to perform an *in vivo* study, 56 male Balb/c mice that had been tumorized by subcutaneous injection of 4T1 cells were divided into eight groups of breast tumor model. Ultrasonic irradiation (US) conditions including intensity of 1.5 W/cm^2^ (with a frequency of 800 kHz, 5 min), MTX concentration of 2 μM, and HGN dose of 2.5 mg/kg (unit of animal weight) were used.

**Results:**

The results show that administration of PEG-HGN-MTX caused a slight reduction in tumor size and growth compared with free MTX. Ultrasound also improved the therapeutic effect of the gold nanoshell in treated groups, and the HGN-PEG-MTX-US treated groups were able to significantly reduce and control tumor size and growth.

**Conclusion:**

The findings also show that MTX and HGN can be used as sonosensitizers in SDT. Also, HGN-PEG-MTX can act as a sono-chemotherapy agent for the combination of sonodynamic therapy and chemotherapy for *in vivo* breast tumors.

## Introduction

Conventional chemotherapy methods cannot effectively destroy cancerous tissue with minimal side effects on healthy tissue. It is not specific to cancer cells and can cause destructive effects in healthy cells as well. Common side effects of chemotherapy include hair loss, nausea, and vomiting. Therefore, a successful chemotherapy method requires a suitable method for drug delivery to cancer cells and less interaction with healthy tissues. Also, using of drug-carrying nanoparticles that have a suitable size for drug delivery to the tumor along with ultrasound waves is considered a developing method of cancer therapy. 

Ultrasound waves are mechanical sounds with continuous vibration frequency above the human hearing range (20 Hz-20 KHz) (1). Low-intensity ultrasound (0.4-2 MHZ) wave is used in non-invasive treatment. Recently, ultrasound wave along with a sonosensitizer called sonodynamic therapy has been used to treat cancer (2). 

Sonodynamic therapy (SDT) is one of the non-invasive promising methods for the eradication of solid cancer tumors. SDT is used to cure deep tumors because ultrasound waves penetrate deeper than other non-invasive methods such as photodynamic therapy (PDT), which uses light instead of ultrasound to activate the photosensitizer (3, 4). There is one other similar non-invasive technique based on ultrasound, named HIFU. It is an alternative method in treating some cancers, especially prostate and breast (5). HIFU commonly has low efficiency and side effect on healthy tissue. So, it is usually used with SDT to use its advantages in the production of reactive oxygen molecules (6).

SDT research has focused on the non-thermal effect of the ultrasound wave. In non-thermal ultrasound, acoustic energy due to the cavitation phenomenon caused pressure on cells (7). Cavitation is a physical phenomenon in which changing of pressure by ultrasound waves in a short time led to the formation of micro-bubbles in tissue fluids. Cavitation occurs in two classes: inertial and noninertial cavitations. In inertial cavitation, the micro-bubbles oscillate, expand and collapse. If the intensity of ultrasound waves is sufficient to cause collapsing of micro-bubbles, sonoluminescence may lead to a sonotoxic effect on the cancer cell. Noninertial cavitation occurs in the presence of a low-intensity acoustic field. And is the process where micro-bubbles are forced to oscillate in presence of the acoustic field when the intensity of the acoustic field is insufficient to collapse the sonosensitizer agent’s micro-bubbles (8). 

Like photosensitizers, which are an important component in photodynamic therapy, sonosensitizers are also the supreme member of sonodynamic therapy, which play an important role in this treatment. These materials are sensitive to ultrasound and are transformed into free radicals due to the heat caused by cavitation, which plays a fundamental role in destroying the tumor (9).

In sonodynamic therapy, following sonosensitizer agents’ uptake by the cells or tissues, after a certain period of time, the treatment site is exposed to ultrasound waves. So, these agents are stimulated and activated by ultrasound and cause cavitation. Eventually, this cavitation leads to the generation of free radicals that results in necrosis and cell death. And in SDT, three elements, low-intensity ultrasound waves, sonosensitizers, and molecular oxygen are required (10). 

Nanomedicines are used to improve the performance of drugs. These nanosystems with sizes 10-100 nm are able to encapsulate drugs that are not soluble and increase their solubility (11). Among the various nanoparticles, gold nanoparticles have attracted much attention for the reason of labeling, delivering, heating, and sensing (12). Gold nanoparticles have been used in medicinal applications due to their unique chemical and physical features, such as low toxicity, surface plasmon resonance, surface modification, and high compatibility (13, 14).

 Hollow gold nanoshells (HGN) can be used as a carrier for chemotherapeutic medications (such as mitoxantrone) to lessen the drug’s cytotoxicity to healthy tissues. It also creates the ideal environment for other processes, such as ultrasonic irradiation (10, 15). HGNs offer unique characteristics such as cavity structure, biological safety, biocompatibility, and high internal and exterior surface availability (16). MTX, in addition to being a chemotherapy drug, also has photosensitization and radio-sensitization capabilities (16, 17). 

In this study, for the first time, the sono-sensitive activity of mitoxantrone was investigated and then MTX was conjugated to hollow gold nanostructure to improve the efficiency of *in vivo* SDT.

## Materials and Methods

***Chemicals***

Mitoxantrone hydrochloride (MW=517.4 g/mol), chloroauric acid (HAuCL4.4H2O), cobalt chloride hexahydrate (99.99%), trisodium dehydrate (>99%), methoxy-polyethylen-glycol (mPEG-SH, MW=2000 g/mol), MTT (3-(4,5 dimethylthiazol-2-yl)-2,5-diphenyltetrazolium bromide), trypan blue, RPMI1640, penicillin, trypsin-EDTA, streptomycin, terephthalic acid, and sodium borohydride (99%) were purchased from Sigma-Aldrich (St. Louis, MO, USA).

***Instrumentations***

The main equipment utilized in this research was a CO_2_ incubator, Elisa reader (AWARENESS, USA), UV-Vis spectrometer (UNICO UV-2100, USA), Dynamic light scattering (DLS) particle size analyzer (Nano-ZS, Malvern, UK), Ultrasound wave generation system (Zimmer, Germany), and Thermovision camera (testo-882, Germany).

***Synthesis of HGN and PEGylating***

**Figure 1:**
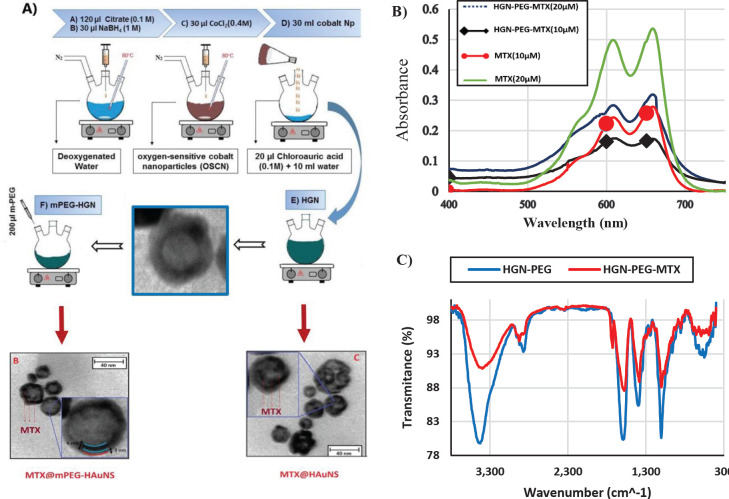
A) Schematic diagram of the steps of synthesis of hollow gold nanoshells, PEGylation process, and conjugation with mitoxantrone, B) UV-Visible spectrophotometry (400-800 nm wavelength) of MTX and HGN-PEG-MTX (m-PEG: 1 mg/ml), C) FT-IR spectra of HGN-PEG and HGN-PEG-MTX

The initial step was synthesizing the oxygen-sensitive cobalt nanoparticles (OSCNs). For this purpose, 120 µl of 0.1 M sodium citrate solution, 30 µl of 1M sodium tetrahydroborate solution, and 30 µl of 0.4 M cobalt chloride solution were added to 30 ml of deoxygenated ultra-pure water respectively with a time interval of one minute. Then a dark brown solution appeared due to the synthesis of OSCNs. After a few minutes, OSCNs were transferred immediately to the vortexing solution, which contained 10 ml deoxygenated ultra-pure water and 20 µl of 0.1 M chloroauric acid. In this step, the gold nanoshells were formed on the surface of the cobalt core. Finally, for removing the core cobalt and oxidization, the solution was exposed to air. To PEGylate hollow gold nanoparticles (HGNs), 200 µl of the methoxy-PEG solution was added to 19.8 ml HGNs solution, and the solution was incubated on the magnet stirrer at room temperature for 4 hr (Figure 1-A) (16).

***Conjugation and release of Mitoxantrone***

**Figure 2:**
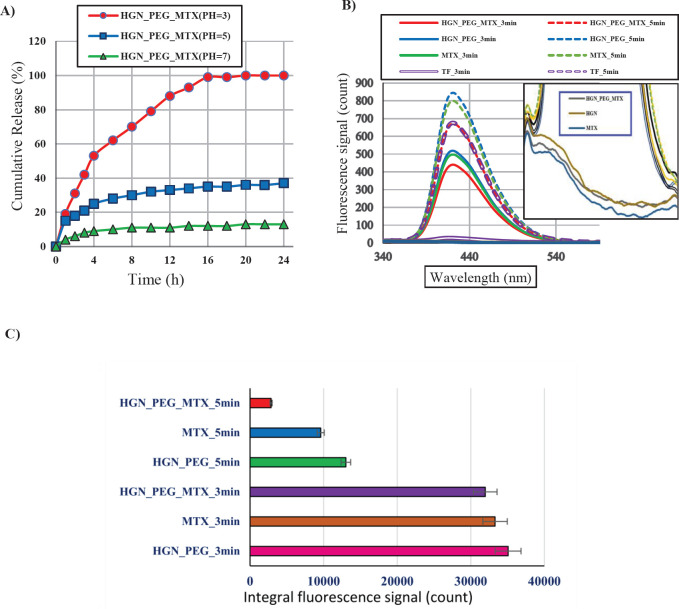
) *In vitro* MTX release study of HGN-PEG-MTX in PBS buffer at different pHs. B) Recorded fluorescence intensity from different modality before removal of the blank signal. C) Integral fluorescence intensity from different modality after removal of the blank signal. D) Experimental set-up of ultrasound exposure

UV-Visible spectrophotometric, spectrofluorometric analyses, and Fourier-transform infrared spectroscopy (FT-IR) were utilized to assess the process of MTX conjugation and release from HGN (Figure 1: B-C, Figure 2: A-C). To investigate the conjugation process, absorption and fluorescence spectra of HGN-PEG-MTX were acquired before, during, and 24 hr after conjugation. Along with the drug release analysis, HGN-PEG-MTX was placed in a quartz cuvette and spectrofluorometric analysis was performed for 24 hr using a Shimadzu spectrofluorometer (Nakagyo-ku, Japan).

***Cell culture process***

4T1 cell line (breast cancer cell line derived from the mammary gland tissue of a mouse BALB/c strain) was obtained from the Pasteur Institute (Tehran, Iran). We used RPMI1640 culture medium to culture cells with 10% fetal bovine serum (FBS) and 100 units/ml penicillin, and 100 µg/ml streptomycin. These cells were kept in the incubator at 5% CO_2_ and adequate humidity at 37 ^°^C. Trypsin-EDTA was used to detach cells from the flask surface and after counting, they were injected.

***Tumor model***

Male BALB/c mice (6-8 weeks and weighting of 20-22 g) were obtained from the Pasteur Institute (Tehran, Iran). The cells (500,0000 cells suspended in 100 µl PBS) were injected subcutaneously into the right flank of BALB/c mice. The mice were housed in the central animal lab of Mashhad University of Medical Science at 23 ^°^C and 65% moisture. After about 2 weeks the tumor reached our interested size. 

***Animal anesthesia***

Before ultrasound wave exposure, the mice were anesthetized by intraperitoneal injection of Ketamine (60 mg/kg) and Xylazine 2% (6 mg/kg) (18) in an animal laboratory.

***Ultrasound generator system***

Mice were exposed to ultrasound waves at the frequency of 0.8 MHz, 1.5 w/cm^2^ intensities in continuous mode for 5 min (Figure 2-D). The diameter of the ultrasonic probe was 2.5 centimeters.

***Experimental protocol of chemical dosimetry***

For preparing the dosimetry solution terephthalic acid (TA) a hydroxyl radical dosimeter, (528 mg TA was dissolved) dissolved in 800 ml deionized water and then treated with 5 ml of 1 M NaOH. The solution was stirred for about 1 hr and kept in a cold (4 ^°^C) and dark place. Figure 2-D shows the experimental set-up of ultrasound exposure. The cylinder contained terephthalic acid. Samples were exposed to ultrasound waves at the frequency of 0.8 MHz, 1.5 w/cm^2^ intensities in continuous mode for 3 and 5 min. The measurements were performed on the groups in Table 1.

***In vitro toxicity of MTX, HGN-PEG, and HGN-PEG-MTX***

The IC_50_ of MTX and HGN-PEG-MTX was determined by using the MTT assay. For doing this, 4T1 cells (mammary gland tissues of mice) were seeded in 96-well plates with a cell density of 7000 cells per well and cultured at a CO_2_ incubator. Then varying concentrations of nanostructures (MTX, HGN-PEG, and HGN-PEG-MTX) that were dissolved in the culture medium were replaced with the culture medium and kept in the incubator. After 90 min incubation, the plates were washed with PBS, and then fresh culture medium (10% FBS) was added to each well. After 24 hr incubation, an MTT assay was performed for each plate. For doing this, 100 µl medium culture without FBS containing 10 µl MTT solution, was added under dark conditions to each well, covered with aluminum foil, and placed inside the incubator. After 4 hr, 200 µl DMSO (dimethyl sulfoxide) was added to dissolve formazan crystals. Then plates were placed on a stirrer with 200 rpm for 5 min and OD (optical density) was read out with an Elisa reader at 570 nm. Cell viability was calculated according to the following equation:

Cell viability(%)=(A_test_-A_blank_)×100/(A_control_-A_blank_)

*(Equation 1)*

***In vivo experiments***

When the average 4T1 tumor volume reached around 120 mm^3^, the tumor-bearing mice were randomly divided into 8 groups (n=7): 1) Control, 2) MTX, 3), HGN-PEG, 4) HGN-PEG-MTX, 5) US, 6)MTX+US, 7)HGN-PEG+US, and 8) HGN-PEG-MTX+US (Table 2). For groups 2, 6 and 3, 7 and 4, 8 and 1, 5, MTX, HGN-PEG and HGN-PEG-MTX, physiological serum was injected into the tumor, respectively. The injective doses of MTX and HGN-PEG were 2 µM and 2.5 mg/kg, respectively. After 10 min of intratumoral injection of the drug, groups 5, 6, 7, and 8 were placed on a plastic cylinder filled with water to match the acoustic impedance so that the tumor side of the mouse was completely in contact with the water. The length of this plastic cylinder was equal to 7 cm, and the tumor of the mouse was placed in the nearby area. Then exposure to ultrasound started.

***Statistical analysis***

Statistical analysis was performed using SPSS software (version 23, USA). Data were analyzed using Kolmogorov-Smirnov, Tukey, one-way ANOVA, and Mann-Whitney test at 95% confidence level.

***Evaluation of treatment efficacy***

The tumor volume (V) and mice weight were measured 30 days after treatment by using a digital caliper with 0.01 mm precision and digital balance with 0.1 g precision respectively every 2 days to evaluate the anti-tumor effects. The tumor volume was measured according to the equation: V=π/6(a×b×c) 

a, b, and c are small diameter, large diameter, and tumor thickness, respectively.

For each tumor, the first day of treatment was considered as day zero (19), and relative tumor volume and relative mice weight on following days were normalized accordingly. In ultrasound-irradiated groups, tumor surface temperature was measured before and after irradiation. Treatment groups were shown in Table 2.

## Results

After preparing of nanoparticles, the size distribution and morphology of the synthesized nanoparticles were characterized using DLS and TEM, respectively. The hydrodynamic size, zeta potential, and polydispersity index were shown in Table 3.

The hydrodynamic sizes of the HGN, HGN-PEG, and HGN-PEG-MTX were measured with DLS, which were 37.8±7 nm, 45.1±8.7, and 63.2±13.9 nm, respectively. The zeta potential of HGN, HGN-PEG, and HGN-PEG-MTX was obtained at -31.4 mv, -19.8 mv, and -37.1 mv, respectively. The particle size of HGN-PEG was increased compared with HGN, and zeta potential of HGN-PEG compared with HGN was increased after PEGylating.

Figure 1-A shows the typical TEM image of the prepared hollow gold nanoparticle. Mitoxantrone loading rates in PEGylated hollow gold nanoparticles were obtained using spectrophotometry. For this purpose, different concentrations of mitoxantrone and methoxy-PEG were used. As shown in Table 4, the percent of mitoxantrone loading in PEGylated hollow nanoparticles was obtained according to the UV-Vis absorbance spectra of MTX, supernatant of HGN-PEG-, and the following formula.

Percent encapsulation=(Total mitoxantrone) -(Mitoxantrone in supernatant)/Total mitoxantrone 

*(Equation 2)*

m-PEG with a concentration of 2 mg/ml was used in this study and the loading percent was considered 40%.

**Figure 3:**
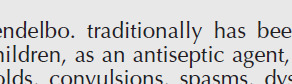
A) Cytotoxicity test of free MTX in 4T1 cells after 90 min incubating. B) Cytotoxicity test of HGN-PEG in 4T1 cells after 90 min incubating. C) Cytotoxicity test of HGN-PEG-MTX in 4T1 cells after 90 min incubating. Data are represented as mean±SD. D) Mean of relative tumors volume until 30 days post-treatment on the different groups. Control, treated with 200 µl physiological serum; MTX, treated with 200 µl MTX at concentration 2 µM; HGN-PEG, treated with 2.5 mg/kg HGN; HGN-PEG+MTX, treated with 2.5 mg/kg HGN and mitoxantrone at concentration 2 µM; US, tumors irradiated with ultrasound wave alone; MTX+US, tumors irradiated with ultrasound wave 10 min after injection of 200 µl MTX at concentration 2 µM; HGN-PEG+US, tumors irradiated with ultrasound 10 min after injection of 2.5 mg/kg HGN; HGN-PEG-MTX , tumors irradiated with ultrasound wave 10 min after injection of 2.5 mg/kg and MTX at concentration 2 µM

Cytotoxicity of free MTX, HGN-PEG, and HGN-PEG-MTX was assessed in 4T1 cells after 90 min incubation. (Figure 3 A-C). According to Figure 3-A significant difference between the control group and all concentrations of MTX (0.1, 0.5, 1, 2.5, and 5 µM) (*P*<0.05). According to Figure 3-B, HGN-PEG has no significant toxicity in 4T1 cells and there were significant differences between the control group and HGN at 93.6, 187.2, and 280.8 mg/l concentrations (*P*<0.05).

According to Figure 3-C, there were significant differences between the control group and MTX at 0.1, 0.5, 1, 2.5, and 5 µM concentrations (*P*<0.05).

Figure 2-A shows *in vitro* drug release study of HGN-PEG-MTX in PBS buffer at different PHs (pH: 3, 5, 7) during 24 hr. The nanostructure was dialyzed under a sterile PBS buffer for 24 hr. At PH=7, after a 10% release of MTX within 4 hr, no significant change was observed in MTX release. As the PH decreased, the MTX release increased significantly. At PH=3 after 15 hr, all of MTX was released within HGN-PEG-MTX.

In order to investigate the amount of hydroxyl radicals produced by the cavitation phenomenon, we used terephthalic acid and ultrasonic radiation with a frequency of 800 kHz, an intensity of 1.5 w/cm^2^, and irradiation times of 3 and 5 min. 

The fluorescence signal generated by the interaction between the hydroxyl radicals and the terephthalic acid was shown in Figure 2: B-C. Results in Figure 2-B indicated that using MTX and HGN-PEG resulted in increased hydroxyl radical generation. And Figure 2-C represents that increasing exposure time will be associated with decreasing in the fluorescence signal (after removing the TA fluorescence signal as blank). We observed that as the irradiation time increased, the production of hydroxyl radicals decreased. The frequency of the fluorescence signal decreased from 3 min to 5 min, and this decrease in fluorescence level in the HGN-PEG-MTX group was significant. The reasons for this phenomenon can be the destruction of gold nanoshells and mitoxantrone drug at high irradiation times, absorption of hydroxyl radicals by mitoxantrone, and increase in the sample temperature with increasing irradiation time and thus decreased sonodynamic properties due to cavitation. Another reason could be some of the ultrasound energy spent to release mitoxantrone from the hollow gold nanoshells. 

Measuring tumor growth during 30 days post-treatment was shown in Figure 3-D. Table 5 shows the Standard Error (SE) of relative tumor volume. There were no inhibitory effects in the control groups. Our results showed that ultrasound wave effects were enhanced in the presence of HGN-PEG (HGN-PEG+US) and HGN-PEG-MTX (HGN-PEG-MTX+US). The synergistic inhibitory effects were significant when HGN-PEG-MTX+US was used. The statistical analysis revealed that there were significant differences found for groups except for the MTX+US group compared with the control (*P*<0.05).

**Figure 4:**
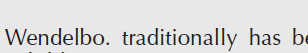
A) Means of doubling time (T2) in the treatment groups (*: *P<*0.001). B) Cumulative survival fractions in the different groups (n=7). Control, treated with 200 µl physiological serum; MTX, treated with 200 µl MTX at concentration 2 µM; HGN-PEG, treated with 2.5 mg/kg HGN; HGN-PEG+MTX, treated with 2.5 mg/kg HGN and mitoxantrone at concentration 2 µM; US, tumors irradiated with ultrasound wave alone; MTX+US, tumors irradiated with ultrasound wave 10 min after injection of 200 µl MTX at concentration 2 µM; HGN-PEG+US, tumors irradiated with ultrasound 10 min after injection of 2.5 mg/kg HGN; HGN-PEG-MTX , tumors irradiated with ultrasound wave 10 min after injection of 2.5 mg/kg and MTX at concentration 2 µM. C) Median survival of mice in different treatment groups

The T2 (amount of time it takes for a tumor to grow to twice its initial size, as measured on the treatment day) of tumor volume in treatment groups is shown in Figure 4-A. The longest and shortest T2 were observed in HGN-PEG-MTX+US (more than 30 days) and MTX+US (8 days), respectively. There were significant differences in T2, between HGN-PEG-MTX+US group with US and HGN-PEG-MTX groups, between the HGN-PEG+US group with US and HGN-PEG groups, between the HGN-PEG-MTX group with Control, and HGN-PEG and MTX groups (*P*<0.001).

Figure 4-B shows the cumulative survival fraction in different groups and Tables 6a, 6b shows additional information about survival. Figure 4-C shows the median survival of mice in different treatment groups. There were no significant differences in median survival between the control group and other groups. But the difference in the median survival between groups of HGN-PEG and HGN-PEG+US, HGN-PEG-MTX and US, and HGN-PEG-MTX and HGN-PEG+US were significant (*P*<0.034).

**Figure 5:**
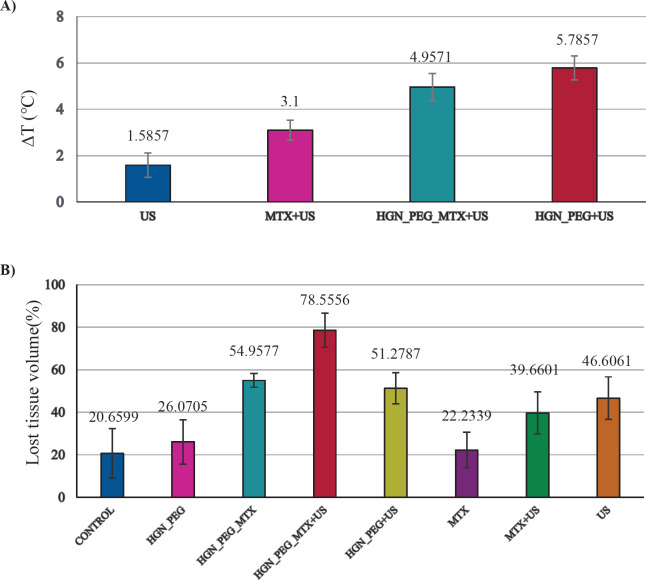
A) Variation of temperature in the treatment groups that exposed ultrasound wave irradiation (0.8 MHz, 1.5 w/cm^2 ^, 5 min). B) Percentage of lost tissue volume in treatment groups. Control, treated with 200 µl physiological serum; MTX, treated with 200 µl MTX at concentration 2 µM; HGN-PEG, treated with 2.5 mg/kg HGN; HGN-PEG+MTX, treated with 2.5 mg/kg HGN and mitoxantrone at concentration 2 µM; US, tumors irradiated with ultrasound wave alone; MTX+US, tumors irradiated with ultrasound wave 10 min after injection of 200 µl MTX at concentration 2 µM; HGN-PEG+US, tumors irradiated with ultrasound 10 min after injection of 2.5 mg/kg HGN; HGN-PEG-MTX , tumors irradiated with ultrasound wave 10 min after injection of 2.5 mg/kg and MTX at concentration 2 µM

HGN: Hollow gold nanoshells; MTX: Mitoxantrone; PEG: Poly ethylene glycol; US: Ultrasound

Figure 5-A shows the temperature variations in the treatment groups that were exposed to ultrasound wave irradiation. As shown in Figure 5-A, HGN-PEG under ultrasound wave irradiation showed a higher variation of temperature in comparison with other groups. In our research, the US group showed significant differences in temperature variations in comparison with other groups (*P*<0.05).

**Figure 6:**
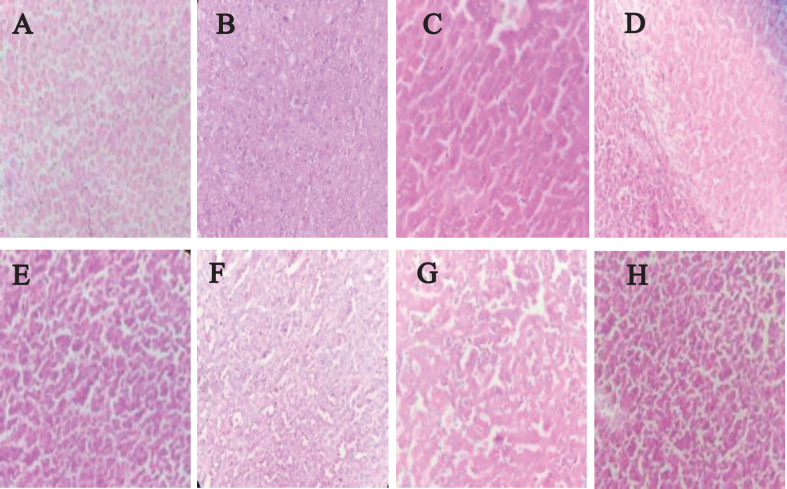
Pathologic specimens collected from tumor slices, demonstrating varying grades of necrotic breast cancer cells in various groups: A) Control, treated with 200 µl physiological serum; B) MTX, treated with 200 µl MTX at concentration 2 µM; C) HGN-PEG, treated with 2.5 mg/kg HGN; D) HGN-PEG+MTX, treated with 2.5 mg/kg HGN and mitoxantrone at concentration 2 µM; E) US, tumors irradiated with ultrasound wave alone; F) MTX+US, tumors irradiated with ultrasound wave 10 min after injection of 200 µl MTX at concentration 2 µM; G) HGN-PEG+US, tumors irradiated with ultrasound 10 min after injection of 2.5 mg/kg HGN; H) HGN-PEG-MTX , tumors irradiated with ultrasound wave 10 min after injection of 2.5 mg/kg and MTX at concentration 2 µM

HGN: Hollow gold nanoshells; MTX: Mitoxantrone; PEG: Poly ethylene glycol; US: Ultrasound

Necrotic cells were seen in all treatment groups based on the pathologic examinations that were conducted (Figure 6).

 The percentage of lost tissue volume in each group is represented in Figure 5-B. According to the results, the largest and lowest amount of lost tissue volume was observed in HGN-PEG-MTX+US (78.5%) and the control group (20.6%), respectively. There were significant differences in lost tissue volume between HGN-PEG-MTX-US group with Control, MTX, and HGN-PEG groups (*P*<0.011).

**Table 1:** Dosimetry grouping with terephthalic acid: 800 kHz (0.8 MHz) ultrasonic frequency and 1.5 w/cm^2^ intensities

Groups	TF	MTX	HGN-PEG	HGN-PEG-MTX
US irradiation time(min)	0	0	0	0
	3	3	3	3
	5	5	5	5

**Table 2:** Treatment groups for in vivo study of Sonodynamic therapy on BALB/c mice

HGN-PEG-MTX	HGN-PEG	MTX	US	Groups
-	-	-	-	**1**
-	-	-	+	**2**
-	-	+	-	**3**
-	+	-	-	**4**
+	-	-	-	**5**
-	-	+	+	**6**
-	+	-	+	**7**
+	-	-	+	**8**

**Table 3:** The characteristics (i.e., particle size, polydispersity index, and zeta potential) of different nanostractures

Nanoparticles	Particle size (nm)	PDI	Conductivity coefficient (mS/cm)	Zeta potential (mv)
HGN	37.8±7	0.274	0.258	-31.4
HGN-PEG	45.1±8.7	0.152	0.165	-19.8
HGN-PEG-MTX	63.2±13.9	0.075	0.110	-37.1

**Table 4:** Percent of Mitoxantrone loading in PEGylated hollow gold nanoparticles for sonodynamic therapy on BALB/c mice

Average	Efficiency	Effective concentration of loading (µM)	Initial concentration (µM)	Nanoparticle
40%	41%	8	20	HGN-PEG(1mg/ml)
	39%	4	10	
41%	40%	8	20	HGN-PEG(2mg/ml)
	42%	4.2	10	

**Table 5:** Standard error of relative tumor volume of BALB/c mice in different treatment groups from the beginning of treatment to one month after treatment

DAY	CONTROL	MTX	HGN-PEG	HGN-PEG-MTX	US	MTX+US	HGN-PEG+US	HGN-PEG-MTX +US
0	0.000	0.000	0.000	0.000	0.000	0.000	0.000	0.000
2	0.111	0.078	0.061	0.108	0.065	0.066	0.077	0.096
4	0.105	0.108	0.066	0.115	0.131	0.212	0.157	0.176
6	0.222	0.142	0.127	0.229	0.137	0.349	0.156	0.263
8	0.185	0.518	0.214	0.231	0.196	0.437	0.171	0.278
10	0.347	0.966	0.148	0.299	0.212	0.501	0.136	0.368
12	0.407	0.955	0.270	0.430	0.279	0.783	0.205	0.424
14	0.430	1.140	0.313	0.485	0.345	0.746	0.263	0.441
16	0.596	1.630	0.343	0.514	0.294	1.021	0.283	0.548
18	0.810	1.398	0.437	0.519	0.308	1.049	0.302	0.608
20	0.793	1.454	0.521	0.909	0.386	1.267	0.346	0.540
22	1.008	1.347	0.531	0.934	0.356	1.213	0.454	0.535
24	0.977	1.294	0.563	0.879	0.359	1.243	0.401	0.536
26	1.097	1.177	0.738	0.941	0.419	1.280	0.373	0.564
28	1.318	1.126	0.722	1.014	0.402	1.414	0.519	0.546
30	1.508	1.066	0.916	1.107	0.384	1.463	0.536	0.546

HGN: Hollow gold nanoshells; MTX: Mitoxantrone; PEG: Poly ethylene glycol; US: Ultrasound

**Table 6:** a) The *P*-value table obtained from the comparison of median survival in different treatment groups in sonodynamic therapy based on the Log-Rank test. b) Overall Comparisons between log rank, breslow, and tarone-ware

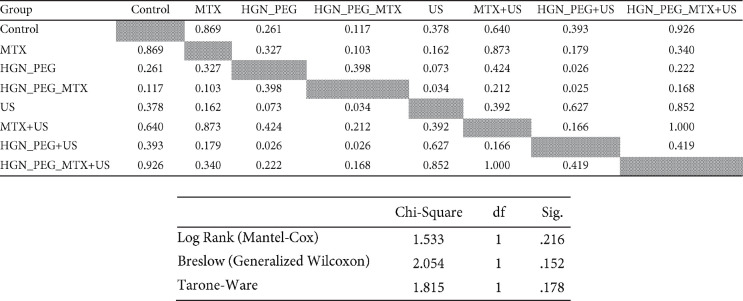

The vector of trend weights is -7, -5, -3, -1, 1, 3, 5, 7. This is the default

HGN: Hollow gold nanoshells; MTX: Mitoxantrone; PEG: Poly ethylene glycol; US: Ultrasound

## Discussion

Chemotherapy, one of the main methods of cancer treatment, plays an important role in fighting cancer. So far, more than 200 anticancer drugs have been used clinically. Studies show that these drugs usually work well in the early stages of the disease, but more than 90% of patients develop drug resistance after relapse (20-22). Due to the low therapeutic index of most chemotherapy drugs, if the tumor is not completely destroyed in the early stages, it can cause drug resistance in the relapse stages of the tumor. Based on these facts, drug resistance has become one of the main obstacles to cancer treatment (22).

So far, researchers have proposed 3 main hypotheses in the field of drug resistance of various types of tumors: (1) Pharmacokinetic effect of the body on the drug: in which the increase in the expression of some membrane proteins leads to the activation of exocytosis systems and drugs pump out of the tumor (23), (2) Genetic mutations of cancer cells: following repeated use of chemotherapy drugs, tumor cells gradually experience genetic mutations and epigenetic changes, which lead to increased resistance of tumors to drugs (24); and (3) The tumor microenvironment (TME), which regulates drug/radiosensitivity of tumor cells and promotes the development of drug-resistant phenotypes (25).

Sonodynamic therapy is an emerging treatment modality that generates reactive oxygen species (ROS) through a combination of low-intensity ultrasound (about 1 MHz) and Sono-sensitizing agents. The main advantage of SDT is that US has a deep penetration of about 10 cm in mammalian tissue, thus making SDT a promising treatment technique for deep tumors (26). Because drug-resistant cells have a higher ROS scavenging rate than sensitive cells, they are more sensitive to ROS. Therefore, the combination of SDT with conventional chemotherapy optimizes therapeutic efficiency, which provides an effective and easy tool to overcome drug resistance.

Mitoxantrone is one of the chemotherapy drugs that cause drug resistance in tumors. It seems that by using combined cancer treatment techniques along with the use of this drug, while reducing the dose and systemic toxicity, its effectiveness can be increased and it can become an exciting topic in research.

The aim of this study was *in vivo* evaluation of the chemo-sonodynamic effect of HGN-PEG-MTX on breast cancer. For this purpose, after tumorizing the animal mice, we used an ultrasound source with a specific frequency and intensity for sonodynamic treatment in the presence of nanocomplexes. In short, in this research after synthesizing the noncomplex, performing the toxicity test on the desired cell line, and comparing the cytotoxicity of MTX, HGN-PEG, and HGN-PEG-MTX groups, we conclude that HGN-PEG does not cause significant toxicity. In contrast, the toxicity of HGN-PEG-MTX is higher than that of the MTX group alone. The release pattern of the drug from the synthesized nanocomplex is evidence that the release rate of mitoxantrone from this nanocomplex is highly dependent on the pH of the environment. In the tumor environment, due to its high pH, we see that it increases with increasing time and reaches its maximum value in 16 hr, but in the normal tissues with a pH of 7, the release of the drug from nanoparticles is very low and does not depend on time, which maintains healthy tissue against the toxic drug mitoxantrone. 

Yoon *et al*. evaluated the toxicity of mitoxantrone-loaded PEGylated Gold nanocomplexes on the HeLa cell line. They also found that toxicity of AuNPs-MTX was more than free MTX, which indicates that these nanostructures can increase the cellular uptake of MTX compared with the free state (15). 

According to the temperature chart in this study, temperature variation during sonodynamic therapy in presence of hollow gold nanoshells was recorded as 5.8 °C, which is not similar to another study by Iraji *et al.* on gold nanoparticles (∆T=7 ^°^C), which can be due to the change of the type of nanoparticles from gold nanoparticles to hollow gold nanoparticles and use low frequencies (800 kHz vs 1 MHz) and low duration of exposure (5 min vs 10 min) in this study (27). 

During the sonodynamic procedure of nanoparticles, the effective factors on thermal phenomena include frequency and irradiation time of ultrasound generator for SDT, and also concentration, shape, size, and conductivity of nanoparticles. Also, PEGylation of nanoparticles causes reduction of conductivity that can generally prevent further temperature rise. In some cases, the agents conjugated to the surface of the nanoparticles can also reduce the conductivity and thus reduce the temperature increase that the nanoparticle alone can produce. 

In this study, the results showed after applying sonodynamic therapy, the HGN-PEG-MTX group attained a lower temperature rise than the HGN-PEG group. This finding can be attributed to the surface modification of HGN with PEG and its conjugation to mitoxantrone that leads to decreased structure conductivity. According to the results of the temperature change curve (Figure 5-A) and tumor dimension change curve (Figure 5-B), because the temperature changes of the hollow gold nanoshell group were greater than the free mitoxantrone group under ultrasound irradiation, the HGN-PEG+US group had better tumor growth control than the MTX+US group. Contrary to the results obtained from the temperature change curve (Figure 5-A) as well as the curve of the produced hydroxyl radicals (Figure 5-B), it was expected that the PEGylated gold nanoshell under ultrasound irradiation would have better tumor growth control. However, according to the relative tumor volume curve (Figure 3-D), the hollow PEGylated gold nanoshell group containing mitoxantrone (the main group) showed a better therapeutic effect under ultrasound irradiation. This reason is related to the chemotherapeutic properties of mitoxantrone, which has a synergistic effect with the thermal and mechanical mechanism of ultrasound and can control tumor growth better. We can see this synergistic effect in many similar articles with different nanoparticle and ultrasound exposure conditions (19, 28). 

Comparison of necrosis results obtained in this study and our previous study was done. It showed that comparing the results of necrosis between this study and our previous study, in which solid gold nanoparticles and ultrasound with a frequency of 1.1 MHz, intensity of 2 watts per square centimeter, and irradiation time of 3 min were used, we can conclude that amount of necrosis of gold nanoshells is higher than solid gold nanoparticles. As a result, hollow gold nanoshells have a better therapeutic effect in the ultrasound field (29). According to the necrosis percentage chart and its noticeable differences between the HGN-PEG-MTX+US group and other groups, we can understand the synergistic effect of chemotherapy, mitoxantrone, and sonodynamic therapy, as well as the sonosensitizing ability of gold nanoshell. The results related to the doubling time of tumor volume also confirm the results related to the percentage of necrosis. According to the results, the maximum time required to double the tumor volume belongs to the group HGN-PEG-MTX+US, which indicates better suppression of the tumor.

## Conclusion

In this study, it was observed that administration of PEGylated hollow gold nanoshells containing mitoxantrone compared with free mitoxantrone increases the cytotoxicity of mitoxantrone on the 4T1 cell line and this effect is directly related to the concentration of mitoxantrone. Dosimetry studies with terephthalic acid also showed that mitoxantrone and PEGylated hollow gold nanoshells have sonosensitizing properties and can be used in sonodynamic therapy. 

The *in vivo* study of 4T1 tumor showed that administration of PEGylated gold nanoshells containing mitoxantrone resulted in a slight reduction in tumor size and growth compared with the free form of mitoxantrone, indicating that hollow gold nanoshells are better carriers for chemotherapeutic drugs such as mitoxantrone.

The prediction based on *in vitro* study, the possibility of increasing mitoxantrone uptake by tumor cells through nanostructure is amplified. Also, the presence of gold hollow nanoshell has intensified the therapeutic effect of ultrasound radiation, so the HGN-PEG-MTX+US treatment group has been able to significantly reduce and control tumor size and growth. This result could indicate the combined effect of sonodynamic therapy and chemotherapy. 

Pathological examination showed that the use of hollow PEGylated gold nanoshells containing mitoxantrone along with ultrasound radiation caused more necrosis in tumor tissue. Based on the findings and observations of this study, it seems that even with the removal of the chemotherapy drug, mitoxantrone, which has several side effects on healthy tissues, and in the presence of hollow gold nanoshells under ultrasound irradiation, the efficiency of sonodynamic therapy can be increased.

## Authors’ Contributions

AS, AI, and BKN designed the experiments; VA, AI, BKN, and HN performed experiments and collected data; AS, AI, BKN, and NTM discussed the results and strategy; AS supervised, directed, and managed the study; AS, AI, and BKN approved the final version to be published.

## Ethical considerations of animal research

The approved code of ethics for animal study at Mashhad University of Medical Sciences is IR.MUMS.MEDICAL.REC.1399.122.

## Conflicts of Interest

All authors declare that they have no conflicts of interest.
